# Application of 3D Gel Dosimetry as a Quality Assurance Tool in Functional Leksell Gamma Knife Radiosurgery

**DOI:** 10.3390/gels8020069

**Published:** 2022-01-20

**Authors:** Linas Kudrevicius, Evelina Jaselske, Diana Adliene, Viktoras Rudzianskas, Andrius Radziunas, Arimantas Tamasauskas

**Affiliations:** 1 Physics Department, Kaunas University of Technology, 44249 Kaunas, Lithuania; linas.kudrevicius@ktu.edu (L.K.); viktoras.rudzianskas@kaunoklinikos.lt (V.R.); 2 Neurosurgery Department, Medical Academy, Lithuanian University of Health Sciences, 44307 Kaunas, Lithuania; andrius.radziunas@kaunoklinikos.lt (A.R.); arimantas.tamasauskas@kaunoklinikos.lt (A.T.); 3 Oncology Institute, Lithuanian University of Health Sciences, 44307 Kaunas, Lithuania

**Keywords:** functional stereotactic radiosurgery, dose verification, QA, gel dosimetry, film dosimetry, Gamma knife, MRI

## Abstract

Highly precise dose delivery to the target (tumor or cancerous tissue) is a key point when brain diseases are treated applying recent stereotactic techniques: intensity-modulated, image-guided radiotherapy, volumetric modulated arc therapy, Gamma knife radiosurgery. The doses in one single shot may vary between tens and hundreds of Gy and cause significant cell/tissue/organ damages. This indicates the need for implementation of quality assurance (QA) measures which are realized performing treatment dose verification with more than one calibrated quality assurance method or tool, especially when functional radiosurgery with a high dose (up to 40 Gy in our case) shall be delivered to the target using small 4 mm collimator. Application of two dosimetry methods: radiochromic film dosimetry using RTQA2 and EBT3 films and dose gel dosimetry using modified nPAG polymer gels for quality assurance purposes in stereotactic radiosurgery treatments using Leksell Gamma Knife© Icon™ facility is discussed in this paper. It is shown that due to their polymerization ability upon irradiation nPAG gels might be potentially used as a quality assurance tool in Gamma knife radiosurgery: they indicate well pronounced linear dose response in hypo-fractionated (up to 10 Gy) dose range and are sensitive enough to irradiation dose changes with a high (at least 0.2 mm) spatial resolution. Dose assessment sensitivity of gels depends on parameters of a dose evaluation method (optical or magnetic resonance imaging), however, is similar to this estimated using film dosimetry, which is set as a standard dosimetry method for dose verification in radiotherapy.

## 1. Introduction

Highly precise dose delivery to the target (tumor or cancerous tissue) is a key point in radiotherapy. This is especially important applying recent stereotactic techniques (IMRT, IGRT, VMAT, Gamma knife radiosurgery) for patient’s treatment since the doses in one single shot may vary between tens and hundreds of Gy and cause significant cell/tissue/organ damages. This indicates the need for implementation of quality assurance (QA) measures which are realized performing treatment dose verification prior to start patient’s irradiation.

**Figure 1:**
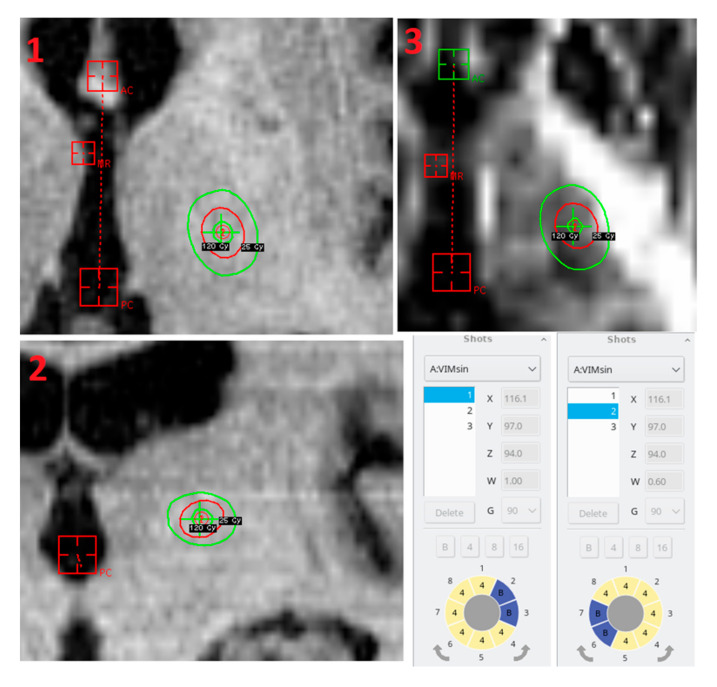
Shot in the shot radiosurgery technique for the left nucleus ventralis intermedius (VIM) thalamotomy, **1**—axial plane, **2**—coronal plane, **3**—MRI tractography (ep2d_diff) axial plane.

Ionization chambers (1D dosimeter) and 2D film dosimetry are usually being used for accurate absorbed dose measurements and dose rate calibration in order to ensure quality control during clinical work of gamma knife stereotactic radiosurgery (SRS) system [1]. Dose rate calibration measurements are usually performed with a special designed Leksell solid water head phantom using PTW 31010 semiflex ionizing chamber or using other instruments and following TG-51 or newer TRS 483 [2] protocol recommendations. However, application of ionization chamber for dose profile estimation requires repeated measurements at different stereotactic locations, thus causing additional positioning uncertainty related to the low spatial resolution. This is in order to increase the measurement accuracy 2D film dosimetry used for dose profile evaluation [3]. It must be noted that before applying film dosimetry in Leksell Gamma Knife machine QA tests, a film calibration (optical density dependency from absorbed dose) is required which accounts for every detail of the gamma knife treatment performance, including treatment environment and devices that are used for minimization of the uncertainties and random errors. A variety of film dosimetry methods are used in radiotherapy, starting with a simple method using scanners [4] to novel methods with wavelength-based approach to increase dose range up to 100 Gy [5]. The results of film exploration for quality checks and quality assurance in external accelerator driven radiotherapy or proton therapy are comparable with those obtained using 2D ionization chamber arrays, imaging plates (IP) and electric portal devices (EPID). However, only film dosimetry from the methods outlined before are suitable for Gamma Knife systems. This is due to a fact that with the increasing radiotherapy machine capabilities, development of the new modalities enabling more precise treatment planning and delivering hypo-fractionated radiosurgery treatments, new requirements for dosimetry systems are set: dosimeters must be able to record dose with a high accuracy and high resolution. Another option for dose assessment in Gamma Knife treatment is polymer gel/ hydrogel dosimetry, which provides 3D dose distribution in almost the same density material as in human soft tissue (e.g., muscle/nPAG = 0.94/1.035) [6]. Polymer gels/hydrogels provide a high accuracy, a good dose resolution, reproducibility, and sensitivity, which depend on irradiation energy and dose rate [7]. Radiation-induced changes of hydrogel’s physical and chemical properties are related to radiation-induced polymerization, which might be assessed experimentally. Since polymerization proceeds in the volume, dose gels can be used for the 3D evaluation of mechanical and geometrical accuracy of Gamma knife shots placement. It can be also used for the comparison and verification of dose calculations for volume irradiation prepared using standard treatment planning system software (Leksell GammaPlan^®^ version 11.1.1 LGP) [8]. This version of treatment planning system performs calculations using a simple water-based algorithm-tissue maximum ratio (TMR 10). Compared to previous TMR, this version includes an additional exponential component μ_0_ (e.g., linear attenuation coefficient of ^60^Co photons traveling the distance between radioactive source and skull surface). Due to a complicated geometrical design of Leksell Gamma Knife^®^Icon™ (Elekta, Stockholm, Sweden) facility (192 ^60^Co sources are arranged in 8 sectors with 24 sources; each source is focused onto single point; collimator positions are foreseen), steep dose gradients are present, thus requesting additional accuracy of dose evaluation in an irradiated volume. Corresponding 3D dose evaluation accuracy can be achieved using gel dosimetry; however, both dose and spatial resolution depends on dosimeter read out technology and requires protocols for dosimeter calibration. This approach comes with the struggle that various dose gel readout methods can be applied: an MRI method which is based on the evaluation of spin-spin relaxation time R2 (1/T2), optical methods, X–ray CT, and ultrasound imaging methods [9]. The number of different gel dosimeter recipes is impressive as well [10,11,12,13]. These range from the Fricke dosimeter, which operates as a radiochromic oxidation-based system [10], to dose gels based on monomers polymerization. Widely used polymer dosimeters may be composed of Methacrylic acid monomers (nMAG gels), Acrylamide with a cross-linker *N*,*N*′-Methylene-bis-acrylamide (nPAG) [11] or *N*-vinylpyrrolidone with a cross-linker *N*,*N*′-Methylene-bis-acrylamide (VIPET) [12]. There are many new derivatives from the the standard recipes created, such as a newly developed PAMPSGAT (Poly AMPS, Gelatin and THPC) gel, in which Acrylamide is replaced by Acrylamide 2-Methyl Propane Sulfonic acid (AMPS) [13] that is less investigated, however, showing their potential as a suitable dosimetry tool for possible application in radiotherapy or brachytherapy. There are even fewer investigations performed regarding the application of gel dosimetry for QA purposes in Gamma Knife radiosurgery. Based on the theoretical suggestions and the scarce experimental information, it seems that polymer gel dosimetry might be successfully used as an independent method for individual treatment plan verification in Gamma Knife Stereotactic Radiosurgery (GK SRS) since very precise dose delivery is required to perform functional radiosurgery, which is applied in the case of trigeminal neuralgia, pain, movement disorders, obsessive-compulsive neurosis, and epilepsy treatment with high doses up to 150 Gy@100% [14,15]. Thalamotomy is one of the examples of radiosurgery procedures applied for the treatment of movement disorders (Figure 1). It is the preferred alternative treatment option for elderly people, with 50–90% tremor reduction because this method is less invasive compared to deep brain stimulation (DBS) [15] targets during radiosurgery procedure, are nucleus ventralis intermedius (VIM) for essential tremor (ET) and pallidothalamic tract (PTT) for tremors caused by Parkinson’s disease (PD) in thalamus. This radiosurgery procedure is realized using a shot-in-the-shot technique (2 shots with the same stereotactic coordinates), delivering the prescribed maximum dose > 130 Gy (dose may vary between 120 and 160 Gy depending on the location of the beam shot center) to the location corresponding to 100% isodose using 4 mm collimator (Figure 1).

VIM and PTT targets are defined by the AC-PC (Anterior commissure—posterior commissure) line in the stereotactic coordinate’s space. Standard AC–PC line is 26 mm long but depending on patient skull anatomy the length can be changed. VIM target is 2–3 mm superior in vertical axis, 6–8.5 mm anterior in axial and 14–18 mm (11 mm from 3rd ventricle wall) in the lateral direction. PTT is 0–2 mm inferior on n vertical axis, 0–2 mm posterior in axial direction and 7.8–12.5 mm in lateral. Maximum dose to internal capsule and to mammillothalamic tract should be minimized and not exceed 19–25 Gy during functional SRS treatment, because the internal capsule consists of white matter structure which separates the caudate and thalamus medially from the putamen and globus pallidus laterally [16]. MRI tractography can help to visualize the internal capsule; however, the patient’s head must be scanned without Leksell^®^ Coordinate Frame G in order to avoid distortions and artefacts. The radiosurgery treatment discussed above would benefit from implementation of an independent dosimetry method, which ensures precise evaluation of the treatment shot location and radiation dose to the internal capsule. Measurements of the mechanical patient positioning system (PPS) accuracy and irradiation plots can be performed using radiochromic films and the results of measurements can be compared with the calculations provided by GK treatment planning system. However, it should be noted that the application of polymer gels for 3D dose distribution evaluation in the internal capsule would provide a more accurate comparison of results regarding GK planned and delivered dose. Furthermore, this dosimetry method would be crucial for the testing and comparison of doses in internal capsule and the total absorbed dose in the thalamus area when different functional SRS techniques are used.

## 2. Results and Discussion

### 2.1. Assessment of Dose Gel Application for Quality Control in Gamma Knife Radiosurgery 

**Figure 2:**
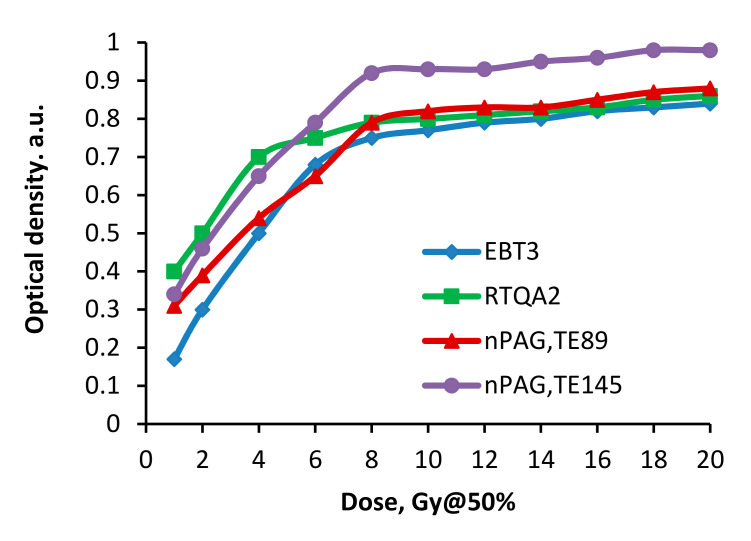
Comparison of dose sensitivity of irradiated polymer gels and radiochomic films.

**Figure 3:**
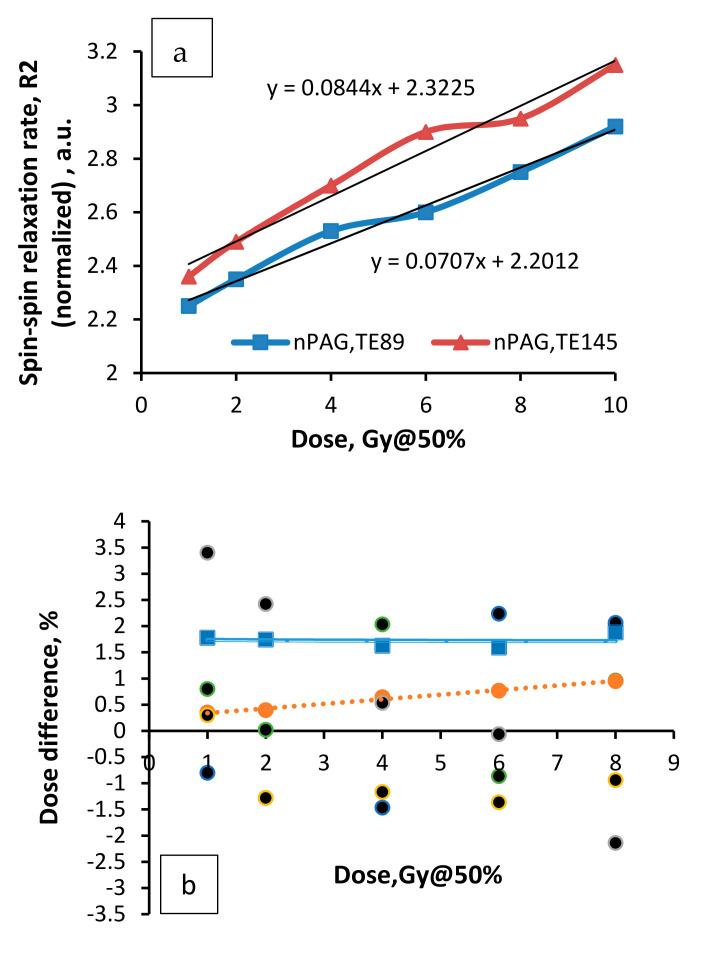
Spin-spin relaxation rate R2 versus absorbed dose curve constructed for Gamma knife irradiated nPAG dose gels (TE 145 ms) (**a**); (**b**) residuals (dashed line) and standard deviations (solid line) of dose measurements.

In our previous investigation [17], we have shown that the selection of echo time (TE) performing MRI scans of irradiated gel samples play an important role in analyzing dosimetry data extracted from DICOM images of irradiated dose gels. In order to assess dose sensitivity of investigated polymer gels irradiated with gamma photons and to verify their feasibility for dosimetry purposes, dose sensitivity curves were constructed using pixel intensity information obtained from MRI scans evaluated using 145 ms and 89 ms echo time sequences. These data were compared with dose sensitivity curves of radiochromic films irradiated in Gamma knife facility, since film dosimetry is a legally approved dosimetry tool for performing quality control procedures in radiotherapy. In both cases pixel intensity was estimated using corresponding software: ImageJ—for evaluation of radiochromic films, and Weasis, for evaluation of MRI scans. This information was used for the construction of corresponding dose sensitivity curves (Figure 2 and Figure 3). It should be noted that prior to start sample irradiation, every absorbed dose value was verified with phantom measurement results obtained using a PTW 30013 ionization chamber. 

Radiochromic films are changing their color due to radiation induced chemical transformations. Taking into account that the evaluation of films was not processed immediately after their irradiation with a high intensity gamma beam, post-irradiation effects leading to some optical saturation of films at higher doses > 8 Gy@50% were possible (Figure 2). A technically limited scanner’s optical sensitivity and scanning mode may also contribute to the deviations of the dose response curve from linear tendency in both irradiated films. 

It was found that the dose response of gamma-irradiated polymer gels was almost linear up to 8 Gy@50% (16 Gy total) and the dose response was slightly dependent on the echo time sequence, which was used for the MRI scans. The estimated dose gel sensitivity to gamma irradiation was 0.082 Gy^−1^ when the 145 ms echo time sequence (TE145) was used for image evaluation and 0.068 Gy^−1^ in the case of 89 ms echo time sequence (TE89) application. Some dose response saturation tendencies were observed at higher doses ≥ 8 Gy@50% indicating that they are very low (0.005 Gy^−1^ for EBT3 and 0.007 Gy^−1^ for RTQA2 films); however, they have a linear dose sensitivity. The sensitivity of radiochromic films was similar to dose gel sensitivity: 0.100 Gy^−1^—for RTQA2 and 0.101 Gy^−1^—for EBT3 films; however, saturation tendency of the dose response curves was observed already at the doses ≥ 4 Gy@50%. RTQA2 films had higher optical density uncertainty values (±0.02–±0.08) as compared to EBT3 (±0.01–±0.03).

Taking into account that an estimation of dose response of irradiated gels, which are based on the evaluation of OD in MRI images is an indirect method and obtained results were verified using primary MRI imaging parameter—spin–spin relaxation rate (R2). The normalized dose response curve constructed using R2 time as a parameter is shown in Figure 3a. Dose uncertainties are demonstrated in Figure 3b: dashed line indicates deviations from the reference values; solid line—standard dose deviations.

Linear dose response of spin-spin relaxation rate (R2) in the dose range between 0 Gy and 10 Gy@50% has been found and this tendency was close to that reported by other authors for basic nPAG gel [18]. The dose sensitivity of nPAG gel estimated using R2 as the main parameter was 0.084 Gy^−1^ and correlated well with the dose sensitivity of the same gel estimated using OD in the MRI image—0.082 Gy^−1^(TE145).

Overall, the performed comparison has shown a similar dose sensitivity using both, gel dosimetry (modified nPAG gel) and radiochromic film dosimetry, thus indicating the ability for both of these methods to be used for Gamma knife radiosurgery.

**Figure 4:**
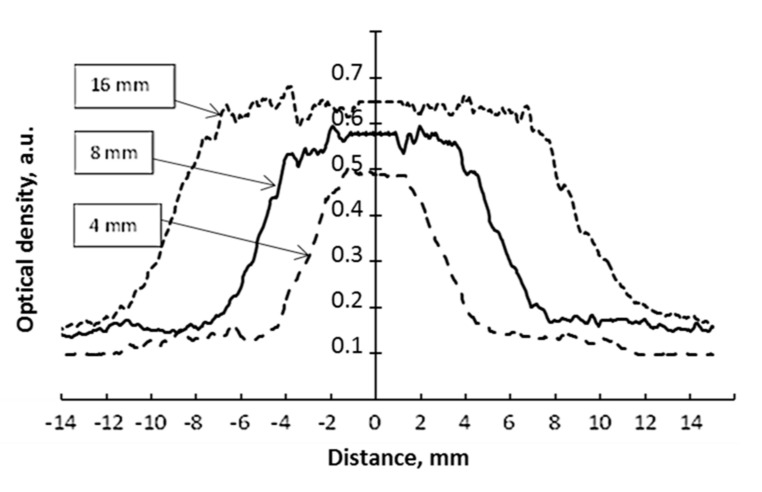
Profiles along the Z axis of nPAG gel samples irradiated with 4 Gy@50% applying differently sized collimators. The collimator size is indicated for each profile separately.

**Figure 5:**
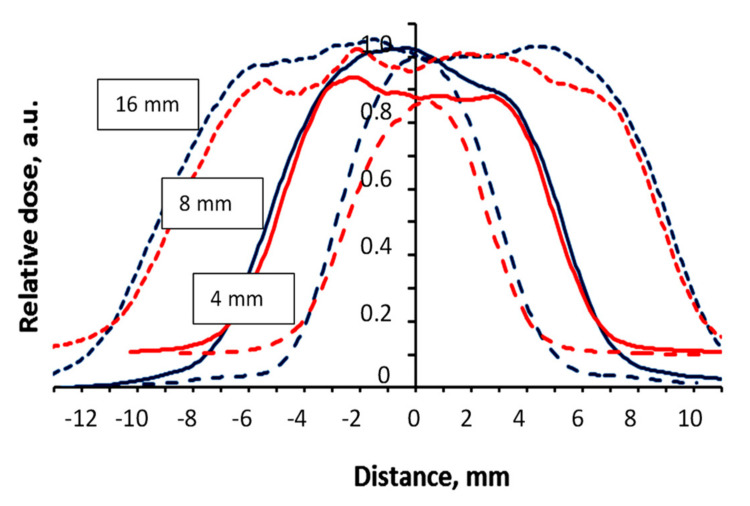
Normalized dose profiles along the Z axis of the RTQA2 (red line) and EBT3 (blue line) films irradiated with 4 Gy@50% dose applying differently sized collimators. Collimator size is indicated for each profile separately.

**Figure 6:**
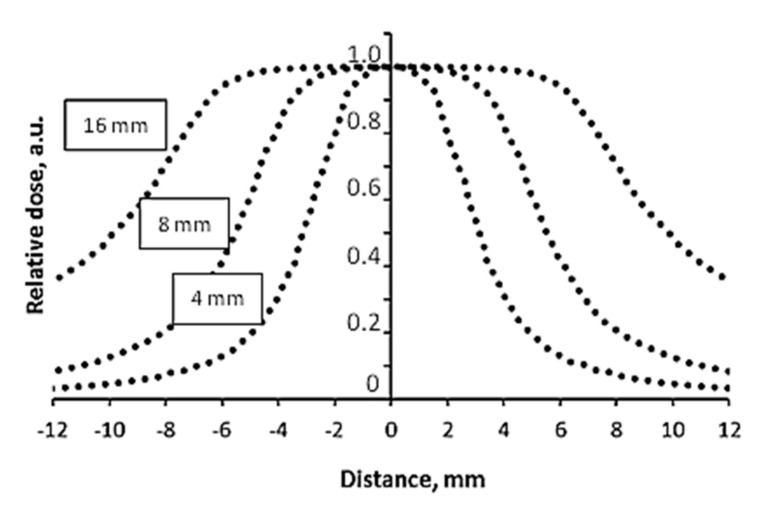
Normalized dose profiles along the Z axis generated by Gammaplan treatment planning system for the 4 Gy@50% dose delivery to the target. Collimator size is indicated for each profile separately.

It should be noted that the geometrical accuracy of dose delivery to the target is also very important in the case of micro-scale treatment, which is used in stereotactic Gamma knife radiosurgery. This issue was thoroughly investigated as well. For this reason, steep dose gradients (0 Gy–4 Gy@50%) were created in nPAG dose gel samples irradiated in the Gamma knife facility, applying differently sized collimators (4 mm, 8 mm, 16 mm). The same conditions were applied for irradiation of radiochromic films. As an example, normalized dose profiles along the Z axis obtained from MRI scans of irradiated nPAG gel samples were created using TE 145 ms, normalized dose profiles of irradiated radiochromic films and dose profiles provided by the Gammaplan treatment planning system (TPS) for differently sized collimators are provided in Figure 4, Figure 5 and Figure 6 respectively.

It was found that the experimentally evaluated dose profiles along the Z axes from irradiated radiochromic films and from reconstructed MRI scans of gels were close to those obtained from TPS-created treatment plans. The linear dependency between the dose and OD, which was found by evaluating radiochromic films and gel’s MRI images after irradaition with the doses from the range up to 8 Gy@50% (Figure 2) was considered when evaluating experimental doses. The obtained experimental dose values were adjusted with the doses measured using a calibrated ionization chamber. Dose measurement uncertainties did not exceed 1%.

Slight differences were observed between the dose profiles obtained evaluating different radiochromic films (RTQA2 and EBT3), which was related to the different sensitivity of both films.

In order to estimate the millimeter scale accuracy of dosimetry methods (films and gels) used for the evaluation of dose delivery in Gamma knife facility, experimentally obtained dose profile values at full width half maximum FWHM (Figure 4 and Figure 5) were compared with the dose profile values along the Z axis, which was provided by the Gamma plan treatment planning system (Figure 6). The FWHM values, together with corresponding uncertainties, are provided in Table 1.

It was found that EBT3 films had a better dynamic range and dose deposition accuracy over RTQA2 films. The difference between experimentally evaluated and simulated FWHM values was varying within the broad interval between 0.3 mm and 1.6 mm for EBT3 films and between 0.3 mm and 1.8 mm for RTQA2 films and was significantly higher compared with the results provided by other authors [18,19]. However, it should be noted that these authors [18,19] were comparing experimental results obtained using a CCD system developed by themselves and the results of EBT3 film measurements, not including theoretical simulations provided by TPS.

Analysis of the results based on MRI evaluation of nPAG gels revealed that the average deviation between the results of FWHM measurements and simulation results was in line with the recommended 5% limit including the highest deviation of 4.2%, which was obtained using an 8 mm collimator. 

Overall, the obtained results indicate a potential of nPAG dose gels for their applications as QA tool in Gamma knife radiosurgery where milli/micro scale tumors are treated. Due to good polymerization ability upon irradiation, these gels are sensitive enough for Gamma knife treatment dose assessment and are characterized by a good spatial resolution (at least 0.2 mm).

### 2.2. Assessment of the Feseability of Dose Gel Application for the Planar Dose Mapping 

Treatment of brain diseases may be realized in single fraction using doses from 10 Gy@50% up to 150 Gy@100%. At least planar (2D) dose distribution in the target is of great importance, since knowledge of it allows for sparring of organs at risk of unnecessary and harmful irradiation. 

To assess the feasibility of dose gel applications for planar dose mapping in radiosurgery, five gel filled vials were irradiated with 10, 13, 18.6, 26 and 43.3 Gy doses, which were determined proportionally according to the Parkinson’s disease treatment plan for a real patient. 

It should be noted that the aim of this pilot investigation was just to assess the suitability of gel dosimetry for dose mapping applications, avoiding absolute evaluation. Application of dose gel filled vials instead of the usual standard cuvettes were considered in order to have more space for indication of dose distribution in irradiated samples, which allowed us to follow the dose-dependent tendencies of shape and intensity changes in polymerized dose gel regions. 

**Figure 7:**
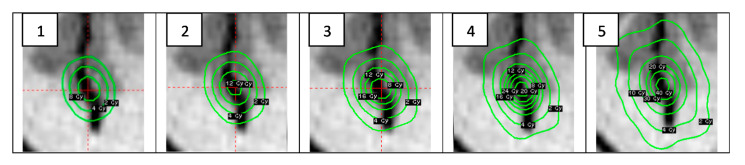
Gammaplan TPS simulated dose distributions with Dmax in the isocenter: **1**—10 Gy, **2**—13 Gy, **3**—18.6 Gy, **4**—26 Gy and **5**—43.3 Gy.

Irradiation was performed in a Gamma knife facility according to the treatment plans simulated using a Gammaplan treatment planning system for functional SRS. Treatment plans were prepared using a TMR 10 algorithm and was visualized using a Sigmaplot 14.0 program package (Figure 7).

**Figure 8:**
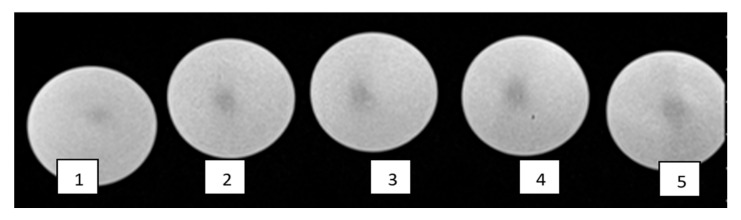
Scanned MRI images of dose gels irradiated with: **1**—10 Gy, **2**—13 Gy, **3**—18.6 Gy, **4**—26 Gy and **5**—43.3 Gy.

Irradiated gels were scanned in a 1.5 T MRI unit by applying the following parameters: FoV (208 × 230), T2 sequence sagittal axis, TE 89 ms and TE 145 ms (Figure 8), TR 4340 ms. Scanned images were analyzed using Weasis v2.5.0 software.

**Figure 9:**
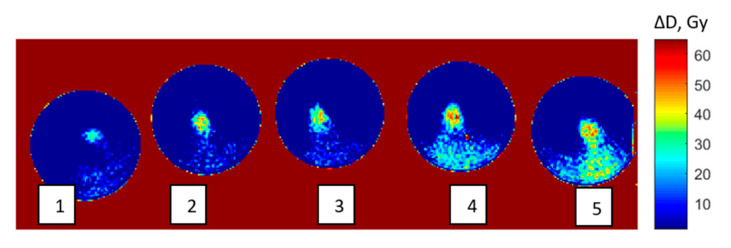
Dose distribution maps of irradiated modified nPAG dose gels created using Matlab programming software: **1**—10 Gy, **2**—13 Gy, **3**—18 Gy, **4**—26 Gy and **5**—43.3 Gy.

Pixel information obtained analyzing MRI scans of irradiated gels was used for the reconstruction of planar dose distributions in the form of dose maps, created by applying special home-developed algorithm written using a MatLab R2016b programing platform. The dose mapping results (Figure 9) allowed for the demonstration of the polymerized volume (volume, which was irradiated) shape changes in the gels, with the increasing dose and for the recording of dose values point by point in irradiated gel. It should be noted that the dose values at certain points were derived from dose calibration curves provided in Figure 2, considering the linearity of the dose response curves in irradiated gels and taking into account that the dose values evaluated using optical methods were verified and corrected using ionization chamber measurement data.

Comparison of the dose values at the selected locations in experimentally created dose maps with the information on the dose values provided by TPS-generated dose distribution maps have shown compatibility of experimental and theoretical results, indicating only small deviations between the data.

**Figure 10:**
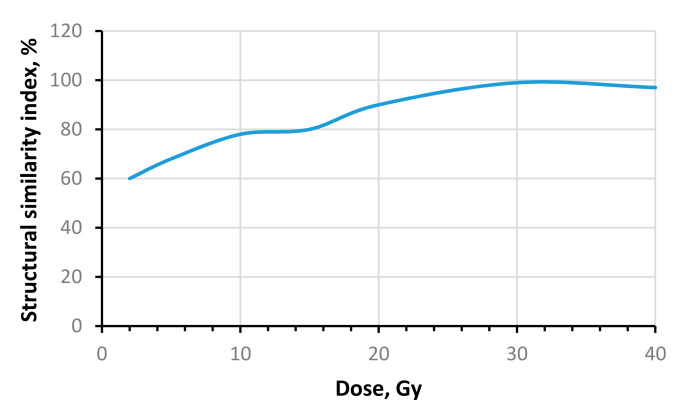
Comparison of dose distribution in irradiated dose gels with a Gammaplan simulated data applying structural similarity index calculations.

For the determination of the similarity of the experimental dose distributions with the isodose profiles provided by TPS Gammaplan, image analysis was performed. A machine-learning-based OpenCV image recognition library was used to avoid possible errors in image matching. After matching, the images were segmented using the Sobel operator, the areas of equal intensity (isodoses) were identified and the structural similarity index (SSIM) for different isodoses was calculated. An example of the obtained similarity results for both dose distributions are shown in Figure 10. It was shown that the similarity of both dose distributions reaches 98% in the radiosurgical dose range and is less pronounced in the radiotherapeutic dose range. The results might be affected by the dose sensitivity and spatial resolution changes in irradiated nPAG gels, which are dependent on different exposure dose ranges (this parameter has not been studied).

A performed pilot investigation indicated the suitability of gel dosimetry for dose mapping applications, avoiding absolute evaluation. As it was shown for the case imitating the real Parkinson disease treatment plan (Figure 7), dose mapping using dose gels in stereotactic radiosurgery is possible and has potential; however, more experimental investigations are needed.

## 3. Conclusions

Assessment of the gel dosimetry method as a possible QA dosimetry tool in functional Leksell Gamma Knife radiosurgery has been performed. Dosimetry data extracted from irradiated nPAG gels was verified with the dosimetry data provided by Gammaplan TPS and was compared with the data evaluated from irradiated radiochromic films, which are recognized as a standard QA dosimetry method in stereotactic radiosurgery. 

Dose response of RTQA2 and EBT3 films was evaluated using open access program ImageJ. It was found that the dose sensitivity was moderate and almost the same for both films (~0.1 Gy^−1^) in the range up to at least 4 Gy@50%, where the linear dose response was observed. At higher doses, some saturation tendencies of optical parameters (dose response) was observed in both films with the increasing dose. This tendency was explained by the fact that the dose delivery in the Gamma knife facility is very intensive and is followed by time dependent post irradiation effects, which contribute to the optical property changes of the exposed films. If dosimetry data are not read-out from films immediately after exposure, optical saturation effects depending on film structure and chemical composition must be considered. 

According to the performed dose delivery accuracy measurements, EBT3 films indicated a higher compliance with dose modelling data; however, the 5% deviation limit for dose delivery accuracy was not exceeded only in the case when 4 mm collimator was used for the irradiation of films.

Dose response of irradiated gels in the range up to 10 Gy@50% was also evaluated by analyzing the MR images, which were obtained at two different echo time sequences: TE 89 ms and TE 145 ms. Two analysis methods were applied: optical, which was realized by analyzing pixel information of MR images using ImageJ program and a method based on the exploration of MRI scanning parameter (e.g., spin–spin relaxation rate, R2). It was found that the dose sensitivity of irradiated dose gels was less, as compared with films and dependent on selected echo time. The estimated dose gel sensitivity to gamma irradiation was 0.084 Gy^−1^ when the 145 ms echo time sequence was used for image evaluation and 0.071 Gy^−1^ in the case of TE 89 ms. The dose sensitivity of 0.082 Gy^−1^ evaluated using Weasis v2.5.0 software directly from MRI scans (R2 vs dose) performed at TE 145 ms was very similar to this obtained using optical method. In both cases, a well pronounced linear dose response was observed in the dose range up to 10 Gy@50%. At higher doses, a linear tendency for saturation was observed. Irradiated dose gels indicated a relatively high accuracy, estimating dose delivery to the spot. The deviations between theoretical FWMH values and experimental (gels) ones did not exceed the 5% limit for all collimators used in the treatment (4 mm, 8 mm, 16 mm), which was related to a good spatial resolution (at least 0.2 mm) required in the verification of brain tumor treatment plans. 

The performed investigations indicated a potential application of nPAG dose gels that are characterized by a relatively high dose sensitivity and spatial resolution as a QA dosimetry tool in Gamma knife radiosurgery where milli/micro scale tumors are treated. 

Moreover, the performed pilot dose mapping experiment has shown that MR images of the irradiated nPAG gels may be used to some extent for planar dose distribution reconstruction. The provided mathematical approach for dose reconstruction (dose mapping) indicated a potential of this method in clinical gel dosimetry applications, especially in radiosurgery using gamma knife treatment where high doses are used to treat small scale objects.

## 4. Materials, Instruments and Methods

### 4.1. Dose Gels

**Figure 11:**
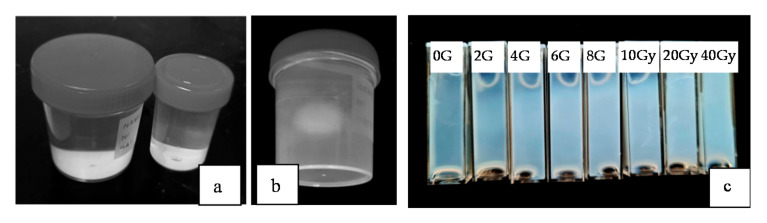
nPAG dose gel filled plastic vials before (**a**) and after irradiation (**b**) in Gamma Knife facility, (**c**) cuvettes filled with nPAG dose gel after irradiation in Gamma knife facility to different doses.

nPAG (Normoxic polyacrylamide) gels were used in this investigation. The selection of nPAG was based on the results of our previous investigations [17,20], which indicated relative high radiation sensitivity, simplicity of manufacturing and broad range of possible modifications, nPAG dose gel was manufactured under normal conditions from 5% gelatine (porcin skin, 300 bloom), 3% Acrylamide (AAm), 3% N,N′-methylene-Bis-acrylamide (BIS), 10 mml (Hydroxymetil) phosphonium chloride with additives (tetrakis, THPC + additives) and 89% ultrapure distilled water. The preparation procedure of the gels was the same as it was described in [21]. Prepared dose gels were poured in six plastic vials, 100 mL each, (Figure 11a,b) and in standard disposable PMMA cuvettes (Figure 11c). The samples were stored in cool and dark place for 24 h to set. During this investigation gels cuvettes with gels from the same batch were irradiated in Leksell Gamma Knife^®^Icon™ (Elekta, Stockholm, Sweden) facility.

### 4.2. Radiochromic Films

**Figure 12:**
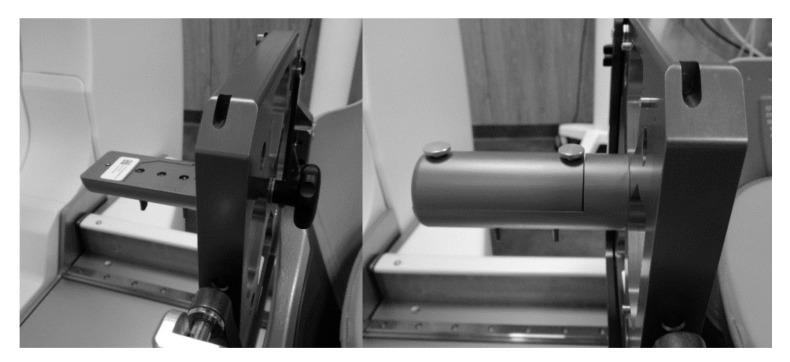
Film holder tool (opened in the left and closed—right).

Taken into account that radiochromic films are widely used in radiotherapy for dosimetry purposes, two types of commonly used films were selected for the verification of gel dosimetry results: EBT3 and RTQA2 films [22]. Films were cut into 25 × 30 mm pieces for irradiation with 4 mm collimator and in 35 × 30 mm pieces for irradiation with 8 and 16 mm collimators and placed at specific locations inside of the film holder (Figure 12). Film holder is calibrated especially for GK QA using films and has a marked point with already known predefined stereotactic space coordinate: (100, 100, 100) mm.

### 4.3. Irradiation of Radiochromic Films

**Figure 13:**
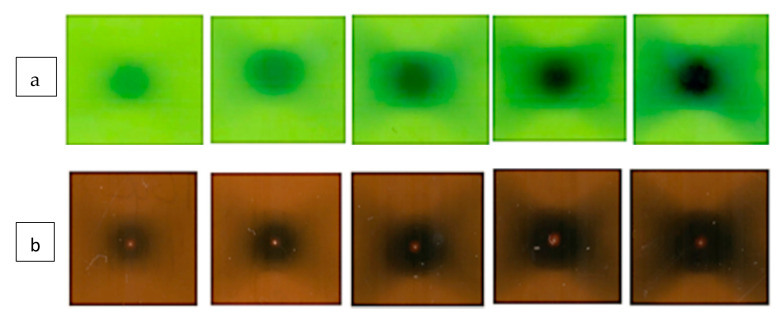
(**a**) Pictures of radiochromic films EBT3 irradiated with different doses (2, 4, 6, 8, 10 Gy@50%) applying 4 mm applicator. (Irradiation doses increases from the left to the right accordingly). (**b**) Pictures of RTQA2 radiochromic films irradiated with different doses (2, 4, 6, 8, 10 Gy@50%) applying 4 mm applicator. (Irradiation doses increases from the left to the right accor-dingly).

The irradiation of films was performed having opened all 8 sectors of sources in Leksell Gamma Knife© Icon™ (GK, Elekta AB, Stockholm, Sweden) facility and delivering 2, 4, 6, 8 and 10 Gy to 50% isodose center (100,100,100) mm with a dose rate of 2.52 Gy/min. Smaller film pieces were irradiated, applying a 4 mm collimator, thus achieving an 0.814 effective output factor, while larger film pieces were irradiated by applying 8 mm and 16 mm collimators with corresponding 0.90 and 1.00 effective outputs. All films were irradiated under the same conditions. After irradiation, all films were scanned by applying open access NAPS2 software, version 6.1.2.25834 (24–bit colour, and 1200 dpi) and were analyzed with open access software ImageJ, version 1.52 p. Examples of scanned radiochromic films are provided in Figure 13.

### 4.4. Irradiation of Dose Gels

The irradiation process of gels was similar to the irradiation of films. It should be noted that the stereotactic coordinate y was changed due to the fact that the dose gel samples were placed at the marked center of the opened film holder tool: y coordinate of 130 mm (for vials) and 110 mm (for cuvettes) instead of 100 mm was used. This allowed us to adjust ^60^Co source focus point at the center of vial or a cuvette filled with polymer gel and reduce positioning uncertainty of the sample.

Experimental cuvettes filled with polymer dose gel were irradiated in Leksell Gamma knife facility having all source sectors opened and applying 16 mm collimator in order to get broader irradiation area with a total dose, 2 Gy, 4 Gy, 6 Gy, 8 Gy, 10 Gy, 20 Gy and 40 Gy. The dose rate of 2.588 Gy/min was applied for sample irradiation.

Five additional nPAG dose gel-filled vials were placed at corresponding locations (100,130,100) mm and irradiated with 10, 13, 18, 26 and 43.3 Gy doses, respectively. Irradiation was performed in a Gamma knife facility according to the treatment plans simulated using a Gammaplan treatment planning system for functional SRS. The irradiation of gels in vials was performed as a pilot project aiming at showing the applicability of dose gels in dose mapping experiments using MRI scans of irradiated samples. However, it was clearly indicated that the evaluation of the absolute dose values needed more detailed investigation.

The delivered doses were checked using an ionization chamber (PTW 30013 ionization chamber), since it is a gold standard for quality control measurements in the Gamma knife facility. According to the international recommendations, the delivered dose calibration using standard dosimetry system was performed twice during the year 2021. In February 2021 the difference between measured and reference dose was −0.21% as well in August 2021 was +0.35%, thus being < 1 %, as it is required in Gamma knife radiosurgery.

We have used an ionization chamber for the verification of the delivered dose prior to each set of experiments. It was found that experimentally delivered dose values differed from the reference values within the interval from −0.26% to +0.35% for the doses up to 10 Gy, and within the interval from −0.21% to 0.26% for the higher doses.

Obtained deviations from the reference values were considered when using experimental dose values for film and gel calibration purposes.

### 4.5. Evaluation of Dose Gels

Dosimetric gels were scanned with MRI Siemens MAGNETOM Avanto 1.5 T unit ~72 h after irradiation. T2 weighted base imaging sequence was registered using a 64 multiple spin-echo pulse sequence and MRI head coil. Two different echo times TE 89 ms and TE 145 ms were selected, since they were found to be most suitable for dose gel imaging [18]. Scanned slice thickness was 2.5 mm, pulse repetition time—4320 s, FoV—453 × 500 and 348 × 512 respectively. Optical densities of dose gels irradiated with different doses were obtained by analyzing pixel intensity values of MRI images.

In order to obtain dose distribution maps in irradiated dose gels, FoV of 208 × 230 was used and transverse relaxation rate (R2) maps of T2 weighted images were calculated using a two point method, which requires a point by point evaluation of signal pixel intensities according to the Equation (1):

1/T2 = R2 = [1/(TE2 − TE1)] × [ln(S(TE1)/S(TE2))](1)

where T2 is relaxation time, R2—calculated transfer relaxation rate, TE—time echo, and S(TE) is signal intensity (pixel value) in the MR image, which was scanned using a corresponding time echo sequence. Calculations were made and acquired images subsequently analyzed using open source software Weasis v2.5.0 and programming platform Matlab R2016b using the method mentioned in our previous publication [17].

## Figures and Tables

**Table 1:** FWHM values and corresponding uncertainties of dose profiles of films and gels irradiated with 4 Gy@50% in Gamma knife facility.

	FWHM, mm (GK Profile, Z Axis)			
	Gammaplan	RTQA2	EBT3	Modified nPAG
4 mm collimator	6.2	5.6	5.9	6.4
Uncertainty		9.7%	4.8%	3.2%
8 mm collimator	11.3	10.4	10.6	11.8
Uncertainty		7.9%	6.6%	4.2%
16 mm collimator	19.8	17.9	18.2	19.5
Uncertainty		10.6%	8.8%	1.5%

## Data Availability

The data in this work are available upon request from the corresponding author.
